# Ex Vivo Expanded Circulating Tumor Cells for Clinical Anti-Cancer Drug Prediction in Patients with Head and Neck Cancer

**DOI:** 10.3390/cancers13236076

**Published:** 2021-12-02

**Authors:** Kuan-Chou Lin, Lai-Lei Ting, Chia-Lun Chang, Long-Sheng Lu, Hsin-Lun Lee, Fang-Chi Hsu, Jeng-Fong Chiou, Peng-Yuan Wang, Thierry Burnouf, Dennis Chun-Yu Ho, Kai-Chiang Yang, Chang-Yu Chen, Chu-Huang Chen, Ching-Zong Wu, Yin-Ju Chen

**Affiliations:** 1 School of Dentistry, College of Oral Medicine, Taipei Medical University, Taipei 110, Taiwan; kclin0628@tmu.edu.tw (K.-C.L.); 99188@w.tmu.edu.tw (D.C.-Y.H.); 2 Department of Oral and Maxillofacial Surgery, Wan Fang Hospital, Taipei Medical University, Taipei 116, Taiwan; 3 Department of Radiation Oncology, Taipei Medical University Hospital, Taipei 110, Taiwan; 971010@h.tmu.edu.tw (L.-L.T.); 123007@h.tmu.edu.tw (L.-S.L.); b001089024@tmu.edu.tw (H.-L.L.); solomanc@tmu.edu.tw (J.-F.C.); 4 Department of Hemato-Oncology, Wan Fang Hospital, Taipei Medical University, Taipei 116, Taiwan; richardch9@tmu.edu.tw; 5 Graduate Institute of Biomedical Materials and Tissue Engineering, College of Biomedical Engineering, Taipei Medical University, Taipei 110, Taiwan; thierry@tmu.edu.tw (T.B.); pumpkin@tmu.edu.tw (K.-C.Y.); 6 International Ph.D. Program in Biomedical Engineering, College of Biomedical Engineering, Taipei Medical University, Taipei 110, Taiwan; 7 TMU Research Center of Cancer Translational Medicine, Taipei Medical University, Taipei 110, Taiwan; 8 Department of Radiology, School of Medicine, College of Medicine, Taipei Medical University, Taipei 110, Taiwan; 9 The Ph.D. Program for Translational Medicine, College of Medical Science and Technology, Taipei Medical University and Academia Sinica, Taipei 110, Taiwan; d622103003@tmu.edu.tw; 10 Department of Chemistry and Biotechnology, Swinburne University of Technology, Melbourne 3122, Australia; pengyuanwang@swin.edu.au; 11 School of Dental Technology, College of Oral Medicine, Taipei Medical University, Taipei 110, Taiwan; 12 Center for Cancer Research, Massachusetts General Hospital, Harvard Medical School, Boston, MA 02129, USA; CACHEN@mgh.harvard.edu; 13 Vascular and Medicinal Research, Texas Heart Institute, Houston, TX 77030, USA; cchen@texasheart.org; 14 Department of Life Innovation, Institute for Biomedical Sciences, Shinshu University, Matsumoto 390-8621, Japan; 15 Department of Dentistry, Taipei Medical University Hospital, Taipei 110, Taiwan; 16 Department of Dentistry, Lo-Tung Poh-Ai Hospital, Yilan 265, Taiwan; 17 Department of Medical Research, Taipei Medical University Hospital, Taipei 110, Taiwan

**Keywords:** circulating tumor cells, drug sensitivity, ex vivo expansion, head and neck cancer, response to therapy

## Abstract

**Simple Summary:**

The conventional methods that seek to predict clinical treatment response are based on the number of circulating tumor cells (CTCs) present in liquid biopsies or genetic profiling of extracted CTCs. This paper presents a novel process by which CTCs can be extracted from blood samples taken from head and neck cancer patients and then expanded ex vivo to form organoids that can be tested with a panel of anti-cancer treatments. The resulting drug sensitivity profiles derived from cisplatin treatment of organoids were subsequently found to correlate with clinical treatment response to cisplatin in patients. CTCs extracted from liquid biopsies for ex vivo expansion negates the need for complicated and potentially risky biopsies of tumor material, thereby supporting the application of this procedure for checkups and treatment monitoring.

**Abstract:**

The advanced-stage head and neck cancer (HNC) patients respond poorly to platinum-based treatments. Thus, a reliable pretreatment method for evaluating platinum treatment response would improve therapeutic efficiency and outcomes. This study describes a novel strategy to predict clinical drug responses in HNC patients by using eSelect, a lab-developed biomimetic cell culture system, which enables us to perform ex vivo expansion and drug sensitivity profiling of circulating tumor cells (CTCs). Forty liquid biopsies were collected from HNC patients, and the CTCs were expanded ex vivo using the eSelect system within four weeks. Immunofluorescence staining confirmed that the CTC-derived organoids were positive for EpCAM and negative for CD45. Two illustrative cases present the potential of this strategy for evaluating treatment response. The statistical analysis confirmed that drug sensitivity in CTC-derived organoids was associated with a clinical response. The multivariant logistic regression model predicted that the treatment accuracy of chemotherapy responses achieved 93.75%, and the area under the curves (AUCs) of prediction models was 0.8841 in the whole dataset and 0.9167 in cisplatin specific dataset. In summary, cisplatin sensitivity profiles of patient-derived CTCs expanded ex vivo correlate with a clinical response to cisplatin treatment, and this can potentially underpin predictive assays to guide HNC treatments.

## 1. Introduction

Head and neck cancer (HNC) is the seventh most common cancer globally, constituting approximately 5.5% of newly diagnosed cancer cases [1]. In GLOBOCAN statistics, HNC showed over 930,000 new cancer cases and more than 460,000 deaths worldwide in 2020, with the highest incidence rates in Melanesia, South Asia, Australasia, Eastern Europe, Western Europe, and North America [1,2]. Key risk factors for HNC include alcohol, tobacco and betel nut use, ultraviolet radiation from sunlight exposure, and human papillomavirus (HPV) infection; specifically, HPV is now the major driving factor of HNC among young adult populations in North America and Europe [2,3]. Overall HNC incidence rates and mortality are expected to continue rising until at least 2060 [2] and this trend has crucial implications for public health, as the economic and healthcare burden of HNC is considerable [4].

For the 30–40% of patients with HNC who present with early-stage (stage I or II) disease at diagnosis, 70–90% of them can achieve disease control and long-term survival from surgery or definitive radiotherapy alone [2,5]. However, over 60% of patients with HNC present with locally-advanced (stage III or IV) disease at diagnosis, with a high risk of local recurrence and distant metastasis [5]. In those advanced HNC patients, cisplatin plays a crucial role in treatment. Surgery followed by adjuvant radiotherapy or concomitant chemoradiotherapy with high-dose cisplatin (100 mg/m^2^) is the standard of care in locally advanced HNC patients with resectable tumors [2,5]. By contrast, concomitant chemoradiotherapy with high-dose cisplatin, administered intravenously once every three weeks for three cycles, is the preferred treatment regimen for those with unresectable tumors [2,5,6]. In addition, TPF (docetaxel, cisplatin, and 5-fluorouracil (5-FU)) induction therapy before concomitant chemoradiotherapy is also a key treatment option [2,5]. However, despite these available treatments, the prognosis for locoregionally-advanced HNC patients remains poor, with 5-year survival rates of less than 50% [5]. Furthermore, the toxicity of chemotherapy and complications usually interrupt therapy—most patients with HNC, especially those receiving radiation or chemoradiation therapy, report experiencing notable symptoms, high pain scores, dysfunction, and poor quality of life [7]. Poor response and resistance to cisplatin is also a vital issue; clinical studies suggest that about 50% of patients do not respond to cisplatin treatment [8], and 65% of those that initially respond eventually develop cisplatin resistance and tumor recurrence [9]. Although studies have sought to identify genetic signatures [8,10] and other predictive factors [11] of cisplatin response, none are clinically applicable as yet.

Circulating tumor cells (CTCs) have been detected in various kinds of epithelial cancer, and the presence of CTCs has been demonstrated to have a clinical impact in cancer patients with metastatic disease [12]. The CTC count is a significant prognostic factor for overall survival in patients with metastatic breast, prostate, colorectal, head, and neck cancer [12,13]. The FDA has approved a method for CTC enumeration used to predict cancer patient outcomes by the CellSearch system [14,15,16]. A detection rate of CTCs ranging from 12–40% has been reported in HNC by Cellsearch system [17,18], which were reported correlated with tumor stage [19], tumor burden [19,20], progression [19,21], lymph node and distant metastasis [20,21,22], recurrence-free survival [20], disease control rate [19,23], progression-free survival [23] and overall survival [23]. However, accurate detection of CTCs has remained a problem; for example, the limitation of the CellSearch system may not capture those CTCs without or with lower epithelial cell adhesion molecules (EpCAM) expression, and CTCs which undergo epithelial-mesenchymal transition have poor capture efficiencies [17,24]. Therefore, different CTC enrichment strategies are used in some studies to evaluate the clinical significance of CTCs in head and neck cancer. For example, Chikamatsu et al. demonstrated the expression level of epithelial marker (KRT19) and mesenchymal marker (vimentin) in CTCs associated with tumor relapse in HNC. Vimentin-positive CTCs, a mesenchymal marker, were higher in recurrent/metastasis patients [25]. Negative selection combined with flow cytometry had been used for CTC isolation; the significant CTC count reduction was found in chemoradiotherapy responders after treatment, which correlated with higher patient survival and one-year recurrence-free rates [18]. In another negative depletion assay, EPISPOT showed that the changes in CTC count correlate with the response to being treated by chemotherapy plus cetuximab in relapsed or metastatic head and neck squamous cell carcinoma [26]. Although CTC enumeration can predict cancer progression and overall survival, other studies show the broad applications of CTCs. The clonal expansion of CTCs could be used to develop an oncology drug screening platform that provides an opportunity to monitor response to therapy noninvasively. Yu et al. showed that CTC cell lines were successfully generated to test the drug sensitivity [27]. However, CTCs are rare, so cell culture from CTCs has become the most challenging unmet need of CTC usages over the past year.

Ex vivo cultures of tumor tissue, cancer stem cells (CSCs), or circulating tumor cells (CTCs) derived from patients purportedly serve as crucial surrogate platforms for assessment of different therapies, thereby enabling clinicians to predict responses for individual patients reliably [12]. Most such studies conducted to HNC thus far have used histocultures of tumor tissue [12,13,14,15,16,17,18] because these tissue sections retained the tumor extracellular matrix and infiltrated host cells, and are reported to preserve the original microenvironment of the tumor well [28]. Drug sensitivity of ex vivo cultures was determined by inhibition rates of 30–40% [29,30], 50% [29,31,32,33], or >50% [29,34], as based on cell survival compared with controls. These studies have demonstrated a strong correlation between cisplatin sensitivity of ex vivo cultures and clinical response to drug treatment [29,30,31,32,33,34], with overall accuracy ranging from 74–78.9%, sensitivity ranging from 71–90.9%, and specificity ranging from 50–78% [29,32,33,34]. However, histocultures are limited due to their very short period of viability of 3–6 days, after which rapid tissue deterioration occurs [28]. A study in 2019 pointed out a potential solution to this problem by extracting tumor cells from patient-derived tumor biopsies or surgical resections to establish patient-derived organoids (PDOs) that could remain viable for an extended period [35]. The study reported culture success rates of 60%, and a correlation between radiosensitivity and clinical response to radiotherapy was noted [35].

Nevertheless, the difficulty of obtaining regular tissue samples to track treatment response or detect resistance limits the application of tumor tissue cultures in a clinical setting. As for CSCs, significant challenges remain in correctly identifying and isolating CSCs from tumor tissue or liquid biopsies. The spheroid cultured from CSCs differs from those in the original tumor [12], limiting their use of surrogates for drug sensitivity testing. In contrast, spheroids cultured from CTCs offer several key advantages. Firstly, CTCs can be extracted from minimally invasive liquid biopsies obtained from the peripheral blood of patients [36], allowing regular sampling to support real-time clinical monitoring of treatment efficacy and resistance development. Secondly, CTCs originate from the primary tumor and are thus histologically and genetically representative [37]. Moreover, studies have already reported ex vivo maintenance of CTCs derived from patients with HNC [38], and organoid cultures expanded ex vivo from CTCs of patients with small cell lung cancer [39]. Tumor organoid cultures are especially suitable for personalized medicine approaches to HNC due to high take rate, fast growth rate, stable morphology and gene expression, a behavior and heterogeneity profile similar to cancer cells in the original tumor, resemblance to the hypoxic tumor microenvironment, and relatively low cost, ease of use, and potential for high-throughput screening [35,40,41]. Therefore, organoid cultures derived from CTCs of patients with HNC can potentially serve as central platforms for real-time assay and prediction of treatment response and resistance in a clinical setting.

In the past, it was difficult to culture CTC in a clinically-relevant time frame reproducibly. As a result, the successful rate of CTC expansion was between 20 and 30% [27,42,43,44]. This study describes a novel process; CTCs were extracted from liquid biopsies of peripheral venous blood from patients with locally advanced HNC and then expanded ex vivo to establish organoid cultures using a lab-developed biomimetic cell culture system, known as eSelect. CTC-derived organoids were subsequently tested with cisplatin, 5-FU, and docetaxel, the most common chemotherapies used to treat locally advanced HNC. Drug sensitivity of CTC-derived organoids was found to correlate with clinical responses in patients with HNC at a 3-month follow-up. Thus, to our knowledge, this is the first study to report the establishment of tumor organoid cultures from CTCs of patients with HNC. It is also the first study to evaluate drug sensitivity using CTC-derived organoids and to correlate the results with actual clinical responses in patients with HNC.

## 2. Materials and Methods

### 2.1. Patient Enrollment

This study was approved by the institutional review board (IRB) of Taipei Medical University Hospital (IRB Number: N201803020, 11 July 2018), and patient samples and data were collected in accordance with the Declaration of Helsinki, Good Clinical Practice, local regulations, and institutional ethical standards. All patients provided written informed consent before study participation. A total of 40 patients with pathologically confirmed and locoregionally advanced head and neck cancer (HNC) were enrolled in this study. According to the American Joint Committee on Cancer (AJCC) Cancer Staging Manual, Eighth Edition [34], the staging was undertaken. Age, sex, tumor staging, alcohol, tobacco, betel nut use, medication information, and clinical response to treatment were evaluated. Patients did not receive any anticancer drugs, radiotherapy, or surgery before liquid biopsy collection. Patients were followed up at three months after liquid biopsy collection, with clinical response to treatment evaluated using computer tomography imaging and Response Evaluation Criteria in Solid Tumors, version 1.1.

### 2.2. CTC Isolation and Expansion

Liquid biopsies of peripheral venous blood (10 mL) were collected from each patient using K2EDTA Vacutainers (BD Bioscience, BD Bioscience, San Jose, CA, USA). A total of 40 liquid biopsies were collected. Liquid biopsies were subjected to Ficoll-Paque centrifugation to isolate the peripheral blood mononuclear cell (PBMC) fraction containing CTCs. CTCs were enriched by RosetteSep™ CTC Enrichment Cocktail kit (Stem cell technologies, Cambridge, MA, USA) as previously described [39]. Cells were seeded onto a binary colloidal crystal (BCC) substrate containing silica and polymethyl methacrylate (PMMA) particles on a culture plate and cultured in DMEM/F12 medium containing EGF, bFGF, B27 supplement and platelet lysate (Thermo Fisher Scientific, Inc., Waltham, MA, USA) for 4 weeks, with culture medium replaced every 4 days [39]. CTC-derived organoids were visualized by microscopy.

### 2.3. Immunofluorescence Staining

The presence of CTCs was confirmed by epithelial cell adhesion molecule (EpCAM) and CD45 immunofluorescence staining, according to a previously-described method [39]. In brief, CTC-derived organoids were collected and resuspended in PBS. The cells were fixed for 10 min using paraformaldehyde, then washed with PBS and followed by subsequently incubated in 0.1% Triton X-100 (in PBS) for permeabilization. Anti-EpCAM (clone EpAb3-5, BioMab, Taipei, Taiwan) and anti-CD45 (clone HI30, Thermo Fisher Scientific, Waltham, MA, USA) primary antibodies were used to confirm the presence of CTCs, and cell nuclei were counterstained using Hoechst 33342. Stained cells were examined under a fluorescence microscope.

### 2.4. Drug Sensitivity Assays

CTC-derived organoids were cultured for 4 weeks, then resuspended in a culture medium and divided into aliquots to be transferred to 96-well culture plates for drug sensitivity assays. A panel of drugs commonly used to treat locally advanced HNC was assessed, including cisplatin, 5-FU, and docetaxel, with tests conducted in triplicate. The clinical pharmacokinetics of each drug were reviewed to determine the appropriate concentration to be applied in drug sensitivity assays [45]. Cells were incubated in each testing drug for 6 days, respectively. Then, viable cell counts for each well were measured using the CellTiter-Glo Luminescent Cell Viability Assay (Promega, Madison, WI, USA) with luminescence read on a GloMax Navigator Microplate Luminometer (Promega, Madison, WI, USA). Relative cell viability was calculated as the percentage of viable cells in the control wells divided by that of the drug-treated wells.

### 2.5. Predictive Model Construction and Selection

A point-biserial correlation test was utilized to evaluate the correlation of relative viability in drug-treated CTC-derived organoids with clinical response in HNC patients. The prognostic model was established by a training dataset that included 75% of overall data, a validation dataset that included all selected samples (total dataset, *n* = 38) and a testing dataset including all cisplatin-treated patients (cisplatin dataset, *n* = 20). Logistic regression analysis was applied to identify the most significant clinical factors from the training data set, remove factors with less significant parameters from univariable analysis, and validate the predictive model with all covariables included in multivariate analysis. A receiver operating characteristic (ROC) curve and Veall-Zimmermann’s Psudo R2 were calculated to compare the optimized model, and the Youden index was used to access the best cutoff value for relative cell viability after drug treatment.

### 2.6. Statistical Analysis

The statistical tests were performed with R package version 3.3.1 (R Core Team, 2018) and GraphPad Prism 7 (San Diego, CA, USA). All analyses performed were two-sided, and statistical significance was determined as two-tailed *p*-values < 0.05.

## 3. Results

### 3.1. Patient Demographics

**Figure 1:**
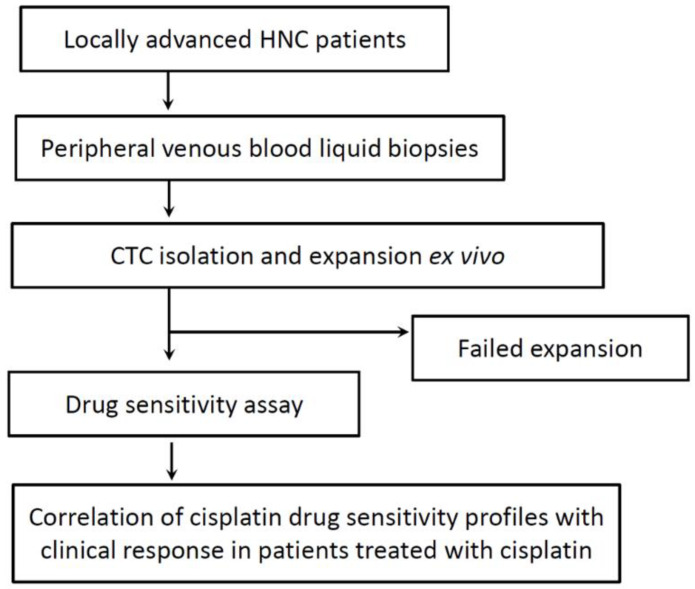
Overview of the study procedure.

A total of 40 patients with locally advanced HNC were enrolled in this study from July 2018 to December 2020 (the patient demographics are detailed in Table 1, and an overview of the study is presented in Figure 1). From these patients, 40 liquid biopsies of peripheral venous blood were collected (the profiles of these liquid biopsies are presented in Table 2). Prior to this, patients received no anticancer drugs, radiotherapy, or surgery.

### 3.2. CTC Expansion and Immunofluorescence Results

**Figure 2:**
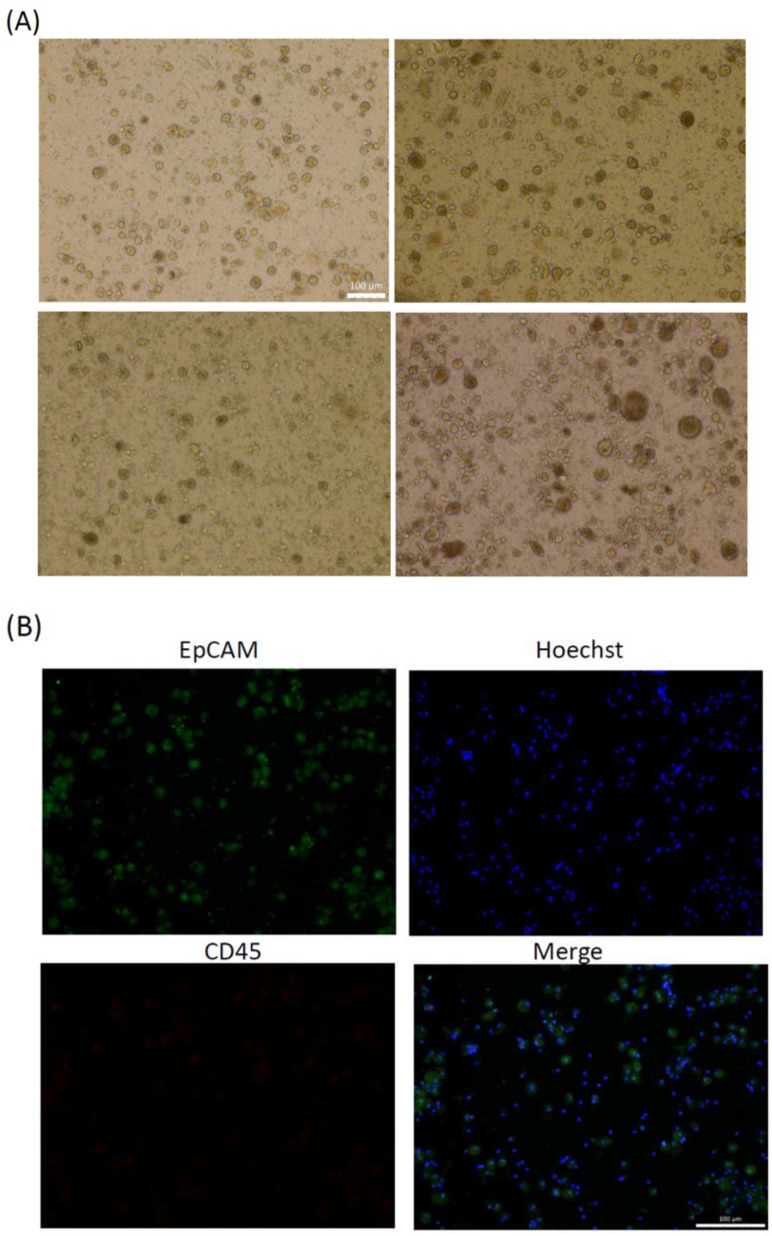
Expansion of ex vivo of circulating tumor cells (CTCs) derived from patients with HNC. (**A**) Representative bright-field images of organoids expanded ex vivo from CTCs derived from patients with HNC. Scale bar: 100 μm. (**B**) Epithelial cell adhesion molecule (EpCAM), or CD45 immunostaining, and Hoechst staining results for organoids to ascertain the presence of CTCs. Scale bar: 100 μm.

Following the isolation of the PBMC fraction from liquid biopsies through Ficoll-Paque centrifugation, fractions containing CTCs were seeded to homemade culture plates. Figure 2A showed that cells proliferated and formed organoids from day 7 to day 28. After 4 weeks of expansion ex vivo, CTC organoids were examined using EpCAM and CD45 immunofluorescence staining, which were defined as being EpCAM-positive and CD45-negative (Figure 2B). Then, CTCs were extracted from the liquid biopsies and expanded ex vivo using the eSelect system. A total of 37 of 40 cultures became successfully established and the ex vivo expansion success rate was 92.50%.

### 3.3. Drug Sensitivity Profiling

The CTC-derived organoids were resuspended and transferred to 96-well culture plates to conduct drug sensitivity assays. The cisplatin, 5-FU, and docetaxel were assayed over six days. Relative viability was calculated as a percentage of surviving cells after test treatment compared with untreated controls, defined as 100%, and lower relative cell viability following application of test treatments indicated superior drug sensitivity and anti-tumor effects. The relative cell viability was established for drug treatment, and the CTC-derived organoids were considered sensitive to that treatment; it was assessed against clinical response to cisplatin treatment. Most patients enrolled in this study had received treatment with cisplatin. Two illustrative case reports that provide insight into the process of drawing correlations and demonstrate the utility of this approach in a clinical setting are presented as follows.

### 3.4. Case Report 1: Complete Response in Stage IV Oral Cancer following Surgery and Cisplatin/Carboplatin Concurrent Chemoradiation Therapy

**Figure 3:**
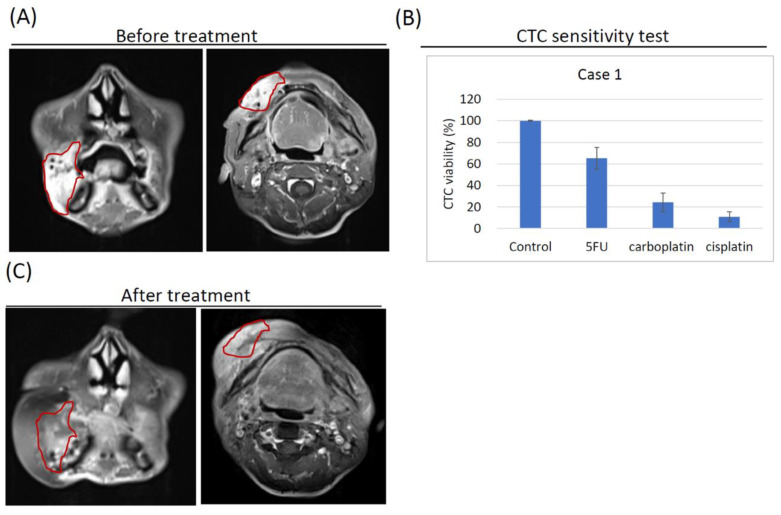

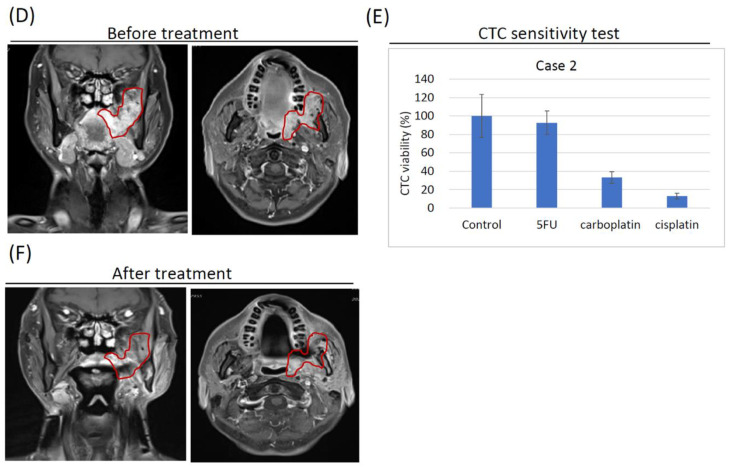
Magnetic resonance imaging (MRI) and eSelect relative cell viability of two illustrative case studies. (**A**) MRI results at diagnosis for Case 1 indicating a raised lesion in the right buccal region with skin induration labelled as the red circle. (**B**) Relative cell viability of CTC-derived organoids treated with 5-FU, carboplatin, or cisplatin by using the eSelect system in Case 1, notably, cisplatin sensitivity was below 20%. (**C**) MRI results at 1 year after concurrent chemoradiation therapy (CCRT) following surgery and adjuvant CCRT with cisplatin and carboplatin for Case 1, red circle represented that complete response to treatment with no residual tumor or lymph node metastasis. (**D**) MRI results at diagnosis for Case 2, the red circle indicated an indurative lesion in the left buccal region that extended to the pterygomandibular space, lateral pharyngeal region, and infratemporal space. (**E**) Relative cell viability of CTC-derived organoids treated with 5-FU, carboplatin, or cisplatin using the eSelect system in Case 2; notably, cells viability was below 20% when exposed to cisplatin. (**F**) MRI results at 1 year after CCRT with cisplatin for Case 2, indicating complete response to treatment with no residual tumor or lymph node metastasis (red circle).

A 46-year-old male patient with a history of alcohol, cigarette and betel nut or quid use was diagnosed with HNC in July 2019. Physical examination revealed a raised ulcerative erythematous lesion of approximately 4.0 × 6.0 × 3.0 cm in the right buccal region, with skin induration noted. Magnetic resonance imaging (MRI) confirmed the location of the lesion (Figure 3A) and indicated that the fat plane was lost between the lesion and the skin. The patient was therefore diagnosed with Stage IVa, mpT4aN0M0 oral cancer (26). CTCs were extracted from a peripheral blood liquid biopsy collected from the patient and subsequently expanded ex vivo. Drug sensitivity assays conducted with the CTC-derived organoids in the eSelect system revealed a relative CTC viability of 65.3 ± 9.9% with 5-FU treatment, 24.3 ± 8.9% with carboplatin treatment, and 11.0 ± 4.6% with cisplatin treatment (Figure 3B). The patient received surgery followed by adjuvant concurrent chemoradiation therapy (CCRT) of 6300 cGy in 35 fractions (fx) together with cisplatin in the 1st and 4th weeks and carboplatin in the 7th week, following National Comprehensive Cancer Network guidelines (5). Regular follow-up and MRI results at one-year post-CCRT indicated that the patient had a complete response to treatment, with no residual tumor or lymph node metastasis observed (Figure 3C).

### 3.5. Case Report 2: Complete Response in Stage IV Oral Cancer following Cisplatin Concurrent Chemoradiation Therapy

A 52-year-old male patient with a history of alcohol use, betel nut or quid chewing, and smoking was diagnosed with HNC in September 2019. Physical examination revealed an indurative and ulcerative erythematous lesion of about 1.5 × 1.5 × 1.0 cm in the left buccal region near tuberosity, with swelling on the left oropharynx also noted. MRI results confirmed the tumor’s location in the left buccal region, extending to the pterygomandibular space, lateral pharyngeal region, and infratemporal space (Figure 3D). A diagnosis of Stage IVb, cT4bN0M0 oral cancer was therefore recorded (26). CTCs from a peripheral blood liquid biopsy collected from the patient were expanded ex vivo, and the CTC-derived organoids were subjected to drug sensitivity assays conducted using the eSelect system. The results showed relative cell viability of 92.5 ± 12.7% with 5-FU treatment, 33.1 ± 6.3% with carboplatin treatment, and 13.0 ± 3.0% with cisplatin treatment (Figure 3E). The patient subsequently received CCRT with 7000 cGy in 35 fx coupled with cisplatin treatment at the 1st, 4th, and 7th weeks. Regular follow-up and MRI results at 1-year post-CCRT revealed a complete response to treatment, with no residual tumor or lymph node metastasis (Figure 3F). Notably, CTCs derived from these two case studies displayed drug sensitivity results below the 20% relative cell viability threshold, indicating cisplatin effectiveness. Both patients achieved a complete response with cisplatin CCRT treatment despite being diagnosed with Stage IV HNC diagnoses. The results highlighted the clinical predictive potential of drug sensitivity assays conducted on CTC-derived organoids using the eSelect system.

### 3.6. Prediction of Clinical Response Based on CTC-Based Drugs Sensitivity Test

**Figure 4:**
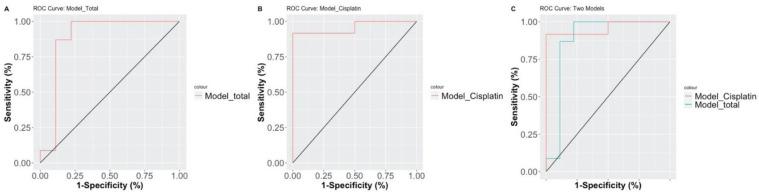
Receiver operating characteristic curves of marker combinations for clinical outcome prediction through eSelect screening as grouped by different data sets. (**A**) Model_Total: a prediction model for the entire data set. (**B**) Model_Cisplatin: prediction model inclusive of all cisplatin-treated subjects; (**C**) Combine Model_Cisplatin and Model_Total.

Participants with complete treatment responses and eSelect drug testing profiles were selected for our logistic regression models to evaluate the prediction accuracy of complete response rates for each head and neck cancer patient. Univariate analysis revealed several parameters associated with clinical response, including stage (*p* = 0.003) and eSelect relative cell viability (*p* = 0.002), and p values reached statistical significance (Table 3). All stepwise-selected factors were applied to multivariate analysis to evaluate the combined effect of multiple variables on clinical responses; stage (odds ratio [OR] = 27.938, 95% confidence interval (CI) = 1537–1642.172, *p* = 0.046) and eSelect relative cell Viability ([OR] = 0.951, 95% CI = 0.899–0.984, *p* = 0.019) were again found to be correlated with clinical response, and the parameter, eSelect relative cell viability, maintains statistical significance (Table 3). The predictive ability of the final multivariate model regarding clinical response was found to show sufficient accuracy (accuracy = 93.75%, chi-square *p* value = 0.00003; Table 3). Area under the curve (AUC) values were applied to compare predictive ability between different parameter combinations, with the final version including eSelect relative cell viability and drug treatment selection. We compared the predictive accuracy of the final logistic regression model through our validation dataset (Model total) and testing dataset (Model Cisplatin). The predictive model demonstrated high accuracy and reliability with an AUC above 0.9 with both datasets (Model total AUC = 0.8841 and Model Cisplatin AUC = 0.9583; Figure 4).

## 4. Discussion

Up to 50% of patients with locally advanced HNC do not respond to cisplatin [8], and 65% of those patients eventually develop resistance to cisplatin [9]. Hence, effective assays that can reliably predict treatment response or provide early detection of treatment resistance, enabling clinicians to provide personalized treatment strategies that enhance outcomes, are urgently required. This study presents an innovative process by which CTCs can be extracted from peripheral blood liquid biopsies of patients with locally advanced HNC and then expanded ex vivo over four weeks to form CTC-derived organoids in a biomimetic cell culture system known as eSelect. The CTC-derived organoids can then be evaluated for their sensitivity to various HNC drug treatments, among which cisplatin, 5-FU, and docetaxel were used in this study. The results showed that the relative cell viability derived from cisplatin sensitivity assays significantly correlated with clinical response to cisplatin treatment in patients with HNC, suggesting that this process can potentially be used in a clinical setting to monitor and guide treatments of patients with HNC.

To our knowledge, this is the first study to report the establishment of tumor organoids by using CTCs derived from peripheral blood liquid biopsies of HNC patients, as well as the first study to deploy CTC-derived organoids expanded ex vivo to assess drug sensitivity and correlate the results with an actual clinical response in HNC patients. CTCs are considered a key prognostic factor in HNC [46,47], and studies have shown that the detection of CTCs in peripheral blood [21,26,48,49,50,51,52,53], high CTC counts in peripheral blood [26], or increasing CTC counts over time [18,26,51,53] were associated with worse prognosis in patients with HNC. Although the US Food and Drug Administration (FDA) has approved the CellSearch system for use in clinical settings to detect and enumerate CTCs in peripheral blood [46,54], merely identifying the presence or number of CTCs does not provide sufficient information to guide treatment decisions. Other studies have sought to provide more detail by assessing the epithelial-mesenchymal status [25,55,56], programmed death-ligand 1 (PD-L1) expression [57], cyclooxygenase-2 expression (COX-2) [58], genetic expression [54], or genomic status [59,60] of CTCs to predict prognosis. However, these studies have not correlated any molecular or genetic characteristics with clinical responses to a specific treatment. This current study followed a different approach, by which CTCs are isolated and expanded ex vivo to generate tumor organoid cultures that can be directly assessed for their sensitivity to cisplatin or other drugs. The paves the way for developing predictive CTC-based assays that can be routinely used to evaluate and monitor treatment response, allowing clinicians to make informed treatment decisions, attain early awareness of treatment resistance, and develop personalized treatment strategies. Patients would also benefit from being treated in ways to which they are more likely to respond, as well as timely notification of resistance and informed guidance about post resistance therapy switching.

Several studies have described the potential of tumor organoid cultures in personalized medicine [40,41,50], and they offer distinct advantages in terms of fast growth, relative stability, a genetic profile similar to that of the original tumor, and capacity for high-throughput screening. The use of PDOs to assess drug sensitivity in HNC has been reported in two studies [41,61], both of which involved cultivating PDOs from biopsied or resected tumor tissue. Both studies indicated that the drug sensitivity of organoids correlated with clinical response to drug treatment in patients [41,61], thereby endorsing the approach presented in the current study. However, the process followed in this study possesses many advantages over those used in previous research. Firstly, the organoid cultures were expanded ex vivo from CTCs extracted from peripheral blood liquid biopsies, which are much easier to obtain than tumor biopsies or resected tumor tissue, especially for locally advanced HNC patients. Liquid biopsies allow real-time drug sensitivity assays to be conducted in a routine clinical setting to predict treatment response, monitor treatment efficacy and detect treatment resistance. These enable the development of a much more flexible, responsive, and effective system of personalized treatment tracking in patients, as opposed to a single one-off test after tumor biopsy or surgery. Secondly, using the eSelect system could achieve CTC extraction and expansion ex vivo from the liquid biopsies of HNC patients within a 4-week period, which is considerably faster than the process described for tumor-tissue derived organoids [41,61] or even for a similar CTC ex vivo expansion study [38]. The eSelect system is thus better positioned to meet the need for quick turnover time and real-time information in actual clinical settings. Thirdly, our study was able to achieve a 92.5% culture success rate using the eSelect system, which is much higher than the 60% [35] and 30.2% [61] expansion rate reported for tumor-tissue derived organoids, or the 28% reported for CTC ex vivo cultures [38]. A high culture success rate is crucial to ensuring the workability of the process in a clinical setting, as low rates would only delay treatment decisions and waste precious time and resources. The utility of the CTC-derived organoid model in this study has previously been assessed in small cell lung cancer [39], and the flexibility, speed, and success rate of this approach are promising for potential future clinical application in the treatment of HNC and other solid tumors.

This study also has some limitations. Firstly, the limited number of patients (*n* = 40) precludes reliable generalization of the results, and more extensive studies are required in the future to validate this process in a larger patient population, specific subtypes of HNC, and different types of treatment. Nevertheless, as indicated by the two case studies presented in the results, this study provided a proof of concept for the eSelect system and the correlation between drug sensitivity results and clinical response, bolstering confidence in this novel approach to be further tested in more extensive future studies. Secondly, this study did not evaluate CTC-derived organoids’ molecular and genetic features reflected in the original tumor in each patient. This was partly due to clinical constraints. As this study included only locally advanced HNC patients, many of them had an unresectable or metastatic disease that prevented further biopsies or surgery from being conducted. However, conducting additional molecular and genetic analyses of CTC-derived organoids to identify biomarkers that could enhance predictive capability accuracy may be beneficial in the future. Moreover, detailed analysis of the molecular or genetic changes in CTC-derived organoids cultured at different stages of treatment would facilitate the monitoring of tumor evolution and resistance development, with important implications for oncology in the future.

## 5. Conclusions

In conclusion, this study developed an efficient and reliable method by which CTCs from liquid biopsies of patients with locally advanced HNC patients can be isolated and expanded ex vivo using the eSelect system. Drug sensitivity assays conducted on the expanded CTC-derived organoids were subsequently correlated with clinical response in patients with HNC. The results highlight the potential of this method to assist clinicians in assessing and predicting treatment response in HNC, and more extensive prospective controlled studies are warranted to validate these findings further and to improve this process for future application in clinical settings.

## Figures and Tables

**Table 1:** Patient demographics.

	Enrolled Patients (*N* = 40)
Mean age, years	57.05
Male, *n* (%)	35 (87.50%)
Female, *n* (%)	5 (12.50%)
Cancer staging	
Stage I–II, *n* (%)	11 (27.50%)
Stage III–IV, *n* (%)	29 (72.5%)
Alcohol use, *n* (%)	14 (35.00%)
Betel nut use, *n* (%)	24 (60.00%)
Smoking, *n* (%)	26 (65.00%)
Treatment	
Cisplatin, *n* (%)	20 (50.00%)
Carboplatin, *n* (%)	7 (17.50%)
5-FU, *n* (%)	7 (17.50%)
Docetaxel, *n* (%)	1 (2.50%)
UFUR, *n* (%)	4 (10.00%)
Other, *n* (%)	12 (30.00%)

UFUR, tegafur uracil.

**Table 2:** Profiles of collected liquid biopsies.

Patient No.	Sex	Age	AJCC Stage	Alcohol	Betel nut	Smoking	CTC Expansion	Viability/Cisplatin	Cisplatin Treatment	Clinical Response
01	M	72	IVA	N	N	N	Y	36.32	N	N/A
02	M	53	IVB	N	N	N	Y	20.37	Y	N/A
03	M	58	IVA	N	N	N	N	58.37	Y	N/A
04	M	46	II	N	Y	Y	Y	107.19	N	CR
05	F	50	IVB	N	N	N	Y	34.72	N	CR
06	M	40	IVA	N	N	Y	Y	11.11	Y	CR
07	F	34	III	N	N	N	N	N	Y	PR
08	M	72	IVA	N	Y	Y	Y	72.85	N	CR
09	M	35	I	N	Y	N	Y	5.27	Y	CR
10	M	58	IVB	N	Y	Y	Y	67.72	Y	N/A
11	M	46	IVB	Y	Y	Y	Y	10.86	N	PD
12	F	81	I	N	N	Y	N	N	N	N/A
13	M	63	I	N	Y	Y	Y	36.58	N	CR
14	M	71	IIB	N	N	N	N	N	N	PD
15	M	53	IVB	N	N	N	Y	43.92	N	PD
16	M	83	IVA	N	Y	Y	Y	9.51	N	CR
17	F	61	IVC	N	N	N	Y	9.45	N	PD
18	M	58	II	Y	Y	Y	Y	4.1	Y	PD
19	M	55	II	N	N	N	Y	5.73	N	CR
20	M	72	III	Y	Y	Y	Y	73.30	Y	PR
21	M	56	IVC	Y	N	N	Y	103.27	N	N/A
22	M	55	IVA	Y	Y	Y	Y	2.60	Y	CR
23	M	56	III	N	N	N	Y	15.87	Y	PR
24	M	46	IVA	Y	Y	Y	Y	12.10	Y	CR
25	F	70	IVA	N	N	N	Y	8.50	N	N/A
26	M	76	IVC	Y	Y	Y	Y	93.22	N	N/A
27	M	52	IVB	Y	Y	Y	Y	13.00	Y	CR
28	M	50	IVB	N	Y	Y	Y	17.60	Y	CR
29	M	56	IVB	N	Y	Y	Y	13.31	N	CR
30	M	63	II	N	Y	Y	Y	122.00	Y	PD
31	M	52	I	N	Y	Y	Y	12.60	N	CR
32	M	43	II	Y	Y	Y	Y	26.80	N	CR
33	M	59	IVB	Y	N	Y	Y	16.90	N	N/A
34	M	48	IVA	Y	Y	Y	Y	21.40	Y	N/A
35	M	50	III	Y	Y	Y	Y	24.50	N	N/A
36	M	80	I	N	Y	Y	Y	10.00	N	N/A
37	M	63	IVA	N	N	N	Y	34.60	N	N/A
38	M	52	III	Y	Y	Y	Y	17.50	Y	CR
39	M	44	IVB	Y	Y	Y	Y	15.10	Y	CR
40	M	49	IVA	N	Y	Y	Y	5.50	Y	CR

N: no, Y: yes, N/A: non-available, CR: complete response, PR: partial response, PD: progressive disease.

**Table 3:** Univariate and multivariate analyses for prognostic variables of clinical outcomes in HNC patients using logistic regression.

Variables	Univariable Analysis			Multivariable Analysis		
	OR	95% CI	*p* Value	OR	95% CI	*p* Value
Age (Years)	0.979	0.914–1.050	0.525			
Stage (I + II vs. III + IV)	21	3.286–204.253	0.003 *	27.398	1.537–1642.172	0.046 *
eSelect cell Viability (<73 vs. >73)	0.954	0.920–0.979	0.002 **	0.951	0.899–0.984	0.019 *

* *p*-value < 0.05, ** *p*-value < 0.01. Accuracy in multivariable model = 93.75%, chi-square *p* value = 0.00003. Multivariable mode: Veall-Zimmermann’s Psudo R^2^ = 0.731, Chi-square *p* value < 0.0001. CI, Confidence Interval; N/A, Not Available; OR, Odds Ratio.

## Data Availability

The datasets from this study are available upon reasonable request to the corresponding author.

## References

[1] Sung H., Ferlay J., Siegel R.L., Laversanne M., Soerjomataram I., Jemal A., Bray F. (2021). Global Cancer Statistics 2020: GLOBOCAN Estimates of Incidence and Mortality Worldwide for 36 Cancers in 185 Countries. CA Cancer J. Clin..

[2] Chow L.Q.M. (2020). Head and Neck Cancer. N. Engl. J. Med..

[3] Aupérin A. (2020). Epidemiology of head and neck cancers: An update. Curr. Opin. Oncol..

[4] Patterson R., Fischman V.G., Wasserman I., Siu J., Shrime M.G., Fagan J.J., Koch W., Alkire B.C. (2020). Global Burden of Head and Neck Cancer: Economic Consequences, Health, and the Role of Surgery. Otolaryngol. Head Neck Surg..

[5] Pfister D.G., Spencer S., Adelstein D., Adkins D., Anzai Y., Brizel D.M., Bruce J.Y., Busse P.M., Caudell J.J., Cmelak A.J. (2020). Head and Neck Cancers, Version 2.2020, NCCN Clinical Practice Guidelines in Oncology. J. Natl. Compr. Cancer Netw..

[6] Oosting S.F., Haddad R.I. (2019). Best Practice in Systemic Therapy for Head and Neck Squamous Cell Carcinoma. Front. Oncol..

[7] Allen-Ayodabo C.O., Eskander A., Davis L.E., Zhao H., Mahar A.L., Karam I., Singh S., Gupta V., Bubis L.D., Moody L. (2019). Symptom burden among head and neck cancer patients in the first year after diagnosis: Association with primary treatment modality. Oral Oncol..

[8] Chang W.-M., Chang Y.-C., Yang Y.-C., Lin S.-K., Chang P.M.-H., Hsiao M. (2019). AKR1C1 controls cisplatin-resistance in head and neck squamous cell carcinoma through cross-talk with the STAT1/3 signaling pathway. J. Exp. Clin. Cancer Res..

[9] Chen Y.-J., You G.-R., Lai M.-Y., Lu L.-S., Chen C.-Y., Ting L.-L., Lee H.-L., Kanno Y., Chiou J.-F., Cheng A.-J. (2020). A Combined Systemic Strategy for Overcoming Cisplatin Resistance in Head and Neck Cancer: From Target Identification to Drug Discovery. Cancers.

[10] Martens-de Kemp S.R., Brink A., Van Der Meulen I.H., De Menezes R.X., Te Beest D.E., Leemans C.R., Van Beusechem V.W., Braakhuis B.J., Brakenhoff R.H. (2017). The FA/BRCA Pathway Identified as the Major Predictor of Cisplatin Response in Head and Neck Cancer by Functional Genomics. Mol. Cancer Ther..

[11] Nakano K., Sato Y., Toshiyasu T., Sato Y., Inagaki L., Tomomatsu J., Sasaki T., Shimbashi W., Fukushima H., Yonekawa H. (2015). Predictive factors of head and neck squamous cell carcinoma patient tolerance to high-dose cisplatin in concurrent chemoradiotherapy. Mol. Clin. Oncol..

[12] Alix-Panabières C., Pantel K. (2013). Circulating Tumor Cells: Liquid Biopsy of Cancer. Clin. Chem..

[13] McMullen K.P., Chalmers J.J., Lang J.C., Kumar P., Jatana K.R. (2016). Circulating tumor cells in head and neck cancer: A review. World J. Otorhinolaryngol. Head Neck Surg..

[14] Hofman V., Ilie M.I., Long E., Selva E., Bonnetaud C., Molina T., Venissac N., Mouroux J., Vielh P., Hofman P. (2011). Detection of circulating tumor cells as a prognostic factor in patients undergoing radical surgery for non-small-cell lung carcinoma: Comparison of the efficacy of the CellSearch Assay™ and the isolation by size of epithelial tumor cell method. Int. J. Cancer.

[15] Riethdorf S., Fritsche H., Müller V., Rau T., Schindlbeck C., Rack B., Janni W., Coith C., Beck K., Jänicke F. (2007). Detection of Circulating Tumor Cells in Peripheral Blood of Patients with Metastatic Breast Cancer: A Validation Study of the CellSearch System. Clin. Cancer Res..

[16] Rossi E., Basso U., Celadin R., Zilio F., Pucciarelli S., Aieta M., Barile C., Sava T., Bonciarelli G., Tumolo S. (2010). M30 Neoepitope Expression in Epithelial Cancer: Quantification of Apoptosis in Circulating Tumor Cells by CellSearch Analysis. Clin. Cancer Res..

[17] Kulasinghe A., Hughes B.G., Kenny L., Punyadeera C. (2019). An update: Circulating tumor cells in head and neck cancer. Expert Rev. Mol. Diagn..

[18] Wang H., Wu M., Chang P., Lin H., Liao C., Wu S., Hung T., Lin C., Chang T., Tzu-Tsen Y. (2019). The change in circulating tumor cells before and during concurrent chemoradiotherapy is associated with survival in patients with locally advanced head and neck cancer. Head Neck.

[19] Buglione M., Grisanti S., Almici C., Mangoni M., Polli C., Consoli F., Verardi R., Costa L., Paiar F., Pasinetti N. (2012). Circulating Tumour Cells in locally advanced head and neck cancer: Preliminary report about their possible role in predicting response to non-surgical treatment and survival. Eur. J. Cancer.

[20] Gröbe A., Blessmann M., Hanken H., Friedrich R.E., Schön G., Wikner J., Effenberger K.E., Kluwe L., Heiland M., Pantel K. (2014). Prognostic Relevance of Circulating Tumor Cells in Blood and Disseminated Tumor Cells in Bone Marrow of Patients with Squamous Cell Carcinoma of the Oral Cavity. Clin. Cancer Res..

[21] Nichols A.C., Lowes L.E., Szeto C.C., Basmaji J., Dhaliwal S., Chapeskie C., Todorović B., Read N., Venkatesan V., Hammond A. (2012). Detection of circulating tumor cells in advanced head and neck cancer using the cellsearch system. Head Neck.

[22] Bozec A., Ilié M., Dassonville O., Long E., Poissonnet G., Santini J., Chamorey E., Ettaiche M., Chauvière D., Peyrade F. (2013). Significance of circulating tumor cell detection using the CellSearch system in patients with locally advanced head and neck squamous cell carcinoma. Eur. Arch. Oto-Rhino-Laryngol..

[23] Grisanti S., Almici C., Consoli F., Buglione M., Verardi R., Bolzoni-Villaret A., Bianchetti A., Ciccarese C., Mangoni M., Ferrari L. (2014). Circulating Tumor Cells in Patients with Recurrent or Metastatic Head and Neck Carcinoma: Prognostic and Predictive Significance. PLoS ONE.

[24] Markiewicz A., Ksiazkiewicz M., Welnicka-Jaskiewicz M., Seroczynska B., Skokowski J., Szade J., Zaczek A.J. (2014). Mesenchymal Phenotype of CTC-Enriched Blood Fraction and Lymph Node Metastasis Formation Potential. PLoS ONE.

[25] Tada H., Takahashi H., Ida S., Nagata Y., Chikamatsu K. (2020). Epithelial–Mesenchymal Transition Status of Circulating Tumor Cells Is Associated With Tumor Relapse in Head and Neck Squamous Cell Carcinoma. Anticancer Res..

[26] Garrel R., Mazel M., Perriard F., Vinches M., Cayrefourcq L., Guigay J., Digue L., Aubry K., Alfonsi M., Delord J.-P. (2019). Circulating Tumor Cells as a Prognostic Factor in Recurrent or Metastatic Head and Neck Squamous Cell Carcinoma: The CIRCUTEC Prospective Study. Clin. Chem..

[27] Yu M., Bardia A., Aceto N., Bersani F., Madden M.W., Donaldson M.C., Desai R., Zhu H., Comaills V., Zheng Z. (2014). Cancer therapy. Ex vivo culture of circulating breast tumor cells for individualized testing of drug susceptibility. Science.

[28] Demers I., Donkers J., Kremer B., Speel E.J. (2020). Ex Vivo Culture Models to Indicate Therapy Response in Head and Neck Squamous Cell Carcinoma. Cells.

[29] Ariyoshi Y., Shimahara M., Tanigawa N. (2003). Study on chemosensitivity of oral squamous cell carcinomas by histoculture drug response assay. Oral Oncol..

[30] Singh B., Li R., Xu L., Poluri A., Patel S., Shaha A.R., Pfister D., Sherman E., Goberdhan A., Hoffman R.M. (2002). Prediction of survival in patients with head and neck cancer using the histoculture drug response assay. Head Neck.

[31] Suzuki H., Nishio M., Hanai N., Hirakawa H., Tamaki T., Hasegawa Y. (2015). Correlation between 18F-FDG-uptake and in vitro chemosensitivity of cisplatin in head and neck cancer. Anticancer Res..

[32] Pathak K.A., Juvekar A.S., Radhakrishnan D.K., Deshpande M.S., Pai V.R., Chaturvedi P., Pai P.S., Chaukar D.A., D’Cruz A.K., Parikh P.M. (2007). In vitro chemosensitivity profile of oral squamous cell cancer and its correlation with clinical response to chemotherapy. Indian J. Cancer.

[33] Hasegawa Y., Goto M., Hanai N., Ijichi K., Adachi M., Terada A., Hyodo I., Ogawa T., Furukawa T. (2007). Evaluation of optimal drug concentration in histoculture drug response assay in association with clinical efficacy for head and neck cancer. Oral Oncol..

[34] Robbins K.T., Connors K.M., Storniolo A.M., Hanchett C., Hoffman R.M. (1994). Sponge-Gel-Supported Histoculture Drug-Response Assay for Head and Neck Cancer: Correlations With Clinical Response to Cisplatin. Arch. Otolaryngol. Head Neck Surg..

[35] Driehuis E., Kolders S., Spelier S., Lõhmussaar K., Willems S.M., Devriese L.A., De Bree R., De Ruiter E.J., Korving J., Begthel H. (2019). Oral Mucosal Organoids as a Potential Platform for Personalized Cancer Therapy. Cancer Discov..

[36] Pantel K. (2019). Circulating Tumor Cells in Head and Neck Carcinomas. Clin. Chem..

[37] Perumal V., Corica T., Dharmarajan A.M., Sun Z., Dhaliwal S.S., Dass C.R., Dass J. (2019). Circulating Tumour Cells (CTC), Head and Neck Cancer and Radiotherapy; Future Perspectives. Cancers.

[38] Kulasinghe A., Perry C., Warkiani M.E., Blick T., Davies A., O’Byrne K., Thompson E.W., Nelson C.C., Vela I., Punyadeera C. (2016). Short term ex-vivo expansion of circulating head and neck tumour cells. Oncotarget.

[39] Lee H.L., Chiou J.F., Wang P.Y., Lu L.S., Shen C.N., Hsu H.L., Burnouf T., Ting L.L., Chou P.C., Chung C.L. (2020). Ex Vivo Expansion and Drug Sensitivity Profiling of Circulating Tumor Cells from Patients with Small Cell Lung Cancer. Cancers.

[40] Yang C., Xia B.-R., Jin W.-L., Lou G. (2019). Circulating tumor cells in precision oncology: Clinical applications in liquid biopsy and 3D organoid model. Cancer Cell Int..

[41] Jin M.-Z., Han R.-R., Qiu G.-Z., Ju X.-C., Lou G., Jin W.-L. (2018). Organoids: An intermediate modeling platform in precision oncology. Cancer Lett..

[42] Alix-Panabières C., Pantel K. (2014). Challenges in circulating tumour cell research. Nat. Rev. Cancer.

[43] Cayrefourcq L., Mazard T., Joosse S., Solassol J., Ramos J., Assenat E., Schumacher U., Costes V., Maudelonde T., Pantel K. (2015). Establishment and Characterization of a Cell Line from Human Circulating Colon Cancer Cells. Cancer Res..

[44] Hernández L.E.C., Eslami-S Z., Alix-Panabières C. (2020). Circulating tumor cell as the functional aspect of liquid biopsy to understand the metastatic cascade in solid cancer. Mol. Asp. Med..

[45] Liston D.R., Davis M. (2017). Clinically Relevant Concentrations of Anticancer Drugs: A Guide for Nonclinical Studies. Clin. Cancer Res..

[46] Habli Z., Alchamaa W., Saab R., Kadara H., Khraiche M.L. (2020). Circulating Tumor Cell Detection Technologies and Clinical Utility: Challenges and Opportunities. Cancers.

[47] Meng Y., Bian L., Zhang M., Bo F., Lu X., Li D. (2020). Liquid biopsy and their application progress in head and neck cancer: Focus on biomarkers CTCs, cfDNA, ctDNA and EVs. Biomark. Med..

[48] Hristozova T., Konschak R., Stromberger C., Fusi A., Liu Z., Weichert W., Stenzinger A., Budach V., Keilholz U., Tinhofer I. (2011). The presence of circulating tumor cells (CTCs) correlates with lymph node metastasis in nonresectable squamous cell carcinoma of the head and neck region (SCCHN). Ann. Oncol..

[49] Tinhofer I., Konschak R., Stromberger C., Raguse J.-D., Dreyer J., Jöhrens K., Keilholz U., Budach V. (2014). Detection of circulating tumor cells for prediction of recurrence after adjuvant chemoradiation in locally advanced squamous cell carcinoma of the head and neck. Ann. Oncol..

[50] Zheng W., Zhang Y., Guo L., Wang S., Fang M., Mao W., Lou J. (2019). Evaluation of therapeutic efficacy with CytoSorter^®^, circulating tumor cell–capture system in patients with locally advanced head and neck squamous cell carcinoma. Cancer Manag. Res..

[51] Liu K., Chen N., Wei J., Ma L., Yang S., Zhang X. (2020). Clinical significance of circulating tumor cells in patients with locally advanced head and neck squamous cell carcinoma. Oncol. Rep..

[52] Rizzo M.I., Ralli M., Nicolazzo C., Gradilone A., Carletti R., Di Gioia C., De Vincentiis M., Greco A. (2020). Detection of circulating tumor cells in patients with laryngeal cancer using ScreenCell: Comparative pre- and post-operative analysis and association with prognosis. Oncol. Lett..

[53] Xun Y., Cao Q., Zhang J., Guan B., Wang M. (2020). Clinicopathological and prognostic significance of circulating tumor cells in head and neck squamous cell carcinoma: A systematic review and meta-analysis. Oral Oncol..

[54] Onidani K., Shoji H., Kakizaki T., Yoshimoto S., Okaya S., Miura N., Sekikawa S., Furuta K., Lim C.T., Shibahara T. (2019). Monitoring of cancer patients via next-generation sequencing of patient-derived circulating tumor cells and tumor DNA. Cancer Sci..

[55] Weller P., Nel I., Hassenkamp P., Gauler T., Schlueter A., Lang S., Dountsop P., Hoffmann A.-C., Lehnerdt G. (2014). Detection of Circulating Tumor Cell Subpopulations in Patients with Head and Neck Squamous Cell Carcinoma (HNSCC). PLoS ONE.

[56] Hsieh J.C., Lin H.C., Huang C.Y., Hsu H.L., Wu T.M., Lee C.L., Chen M.C., Wang H.M., Tseng C.P. (2015). Prognostic value of circulating tumor cells with podoplanin expression in patients with locally advanced or metastatic head and neck squamous cell carcinoma. Head Neck.

[57] Strati A., Koutsodontis G., Papaxoinis G., Angelidis I., Zavridou M., Economopoulou P., Kotsantis I., Avgeris M., Mazel M., Perisanidis C. (2017). Prognostic significance of PD-L1 expression on circulating tumor cells in patients with head and neck squamous cell carcinoma. Ann. Oncol..

[58] Li Y.-J., Luo Y., Xie X.-Q., Li P., Wang F. (2018). The prognostic value of COX-2 expression on circulating tumor cells in nasopharyngeal carcinoma: A prospective analysis. Radiother. Oncol..

[59] Tada H., Takahashi H., Kawabata-Iwakawa R., Nagata Y., Uchida M., Shino M., Ida S., Mito I., Matsuyama T., Chikamatsu K. (2020). Molecular phenotypes of circulating tumor cells and efficacy of nivolumab treatment in patients with head and neck squamous cell carcinoma. Sci. Rep..

[60] Tada H., Takahashi H., Kuwabara-Yokobori Y., Shino M., Chikamatsu K. (2020). Molecular profiling of circulating tumor cells predicts clinical outcome in head and neck squamous cell carcinoma. Oral Oncol..

[61] Tanaka N., Osman A.A., Takahashi Y., Lindemann A., Patel A.A., Zhao M., Takahashi H., Myers J.N. (2018). Head and neck cancer organoids established by modification of the CTOS method can be used to predict in vivo drug sensitivity. Oral Oncol..

